# A Review on Tumor Control Probability (TCP) and Preclinical Dosimetry in Targeted Radionuclide Therapy (TRT)

**DOI:** 10.3390/pharmaceutics14102007

**Published:** 2022-09-22

**Authors:** Kaat Spoormans, Melissa Crabbé, Lara Struelens, Marijke De Saint-Hubert, Michel Koole

**Affiliations:** 1 Research in Dosimetric Applications, Belgian Nuclear Research Center (SCK CEN), 2400 Mol, Belgium; 2 Unit of Nuclear Medicine and Molecular Imaging, Department of Imaging and Pathology, Katholieke Universiteit Leuven (KUL), 3000 Leuven, Belgium; 3 NURA Research Group, Belgian Nuclear Research Center (SCK CEN), 2400 Mol, Belgium

**Keywords:** tumor control probability, targeted radionuclide therapy, dosimetry, preclinical, dose rate, heterogeneity, RBE, proliferation

## Abstract

Targeted radionuclide therapy (TRT) uses radiopharmaceuticals to specifically irradiate tumor cells while sparing healthy tissue. Response to this treatment highly depends on the absorbed dose. Tumor control probability (TCP) models aim to predict the tumor response based on the absorbed dose by taking into account the different characteristics of TRT. For instance, TRT employs radiation with a high linear energy transfer (LET), which results in an increased effectiveness. Furthermore, a heterogeneous radiopharmaceutical distribution could result in a heterogeneous dose distribution at a tissue, cellular as well as subcellular level, which will generally reduce the tumor response. Finally, the dose rate in TRT is protracted, relatively low, and variable over time. This allows cells to repair more DNA damage, which may reduce the effectiveness of TRT. Within this review, an overview is given on how these characteristics can be included in TCP models, while some experimental findings are also discussed. Many parameters in TCP models are preclinically determined and TCP models also play a role in the preclinical stage of radiopharmaceutical development; however, this all depends critically on the calculated absorbed dose. Accordingly, an overview of the existing preclinical dosimetry methods is given, together with their limitation and applications. It can be concluded that although the theoretical extension of TCP models from external beam radiotherapy towards TRT has been established quite well, the experimental confirmation is lacking. Thus, requiring additional comprehensive studies at the sub-cellular, cellular, and organ level, which should be provided with accurate preclinical dosimetry.

## 1. Introduction

Ever since the discovery of ionizing radiation at the end of the 19th century, it has been exploited for various medical applications. One of them is cancer treatments, where the aim is to deliver a high absorbed dose to the tumor, while minimizing the radiation burden to healthy tissue. Targeted radionuclide therapy (TRT) is a relatively novel approach, where a radionuclide can be coupled to well-designed vectors who specifically target the cancerous cells. These so-called radiopharmaceuticals (RP) are administered systemically, and thus, are able to target cancerous cells throughout the whole body, such that inoperable or metastasized tumors can be treated.

The emission of short-range alpha, beta or Auger electron radiation by specific radionuclides causes DNA damage within the cancerous cells, thereby inducing cell death by a very localized dose deposition. Although approved radiopharmaceutical treatment schedules usually follow the ’one size fits all’ approach, each patient may display a different treatment response based on tumor volume, radiosensitivity of tumor cells, biokinetics of the RP, and the repair mechanisms of tumor cells. Individualized treatment planning is currently not yet a standard practice of care in TRT, but may significantly improve therapeutic efficacy. Personalized treatment would benefit from a mathematical model that is able to predict the treatment outcome for a specific patient and a given absorbed dose. From external beam radiotherapy (EBRT), there is the Tumor Control Probability (TCP) model with TCP = 0 corresponding to no response expected, while TCP = 1 corresponds to a 100% chance that all cancerous cells will be killed and the patient is cured. The general TCP model accounts for the overall absorbed dose and the radiosensitivity of tumor tissue, while it makes a distinction between repairable, sub-lethal DNA single strand breaks (SSB) and unrepairable, lethal DNA double strand breaks (DSB), as will be explained in Section 2. More extensive models also incorporate the effects of tumor repopulation, tumor heterogeneity, differences in dose rate and linear energy transfer (LET), and heterogeneous dose distributions, of which the latter three are especially relevant in TRT.

For EBRT, an abundance of data have been collected in recent decades, which resulted in well-established TCP models [1]. A direct translation to the more novel TRT is, however, not straightforward, since their irradiation characteristics differ substantially. Unlike EBRT, TRT results in an absorbed dose that is heterogeneously distributed across the tumor, both on a tissue and subcellular level, while the dose rate is protracted, relatively low and variable over time. Furthermore, TRT exploits high and medium LET alpha, beta, or Auger electron radiation, while in EBRT, low LET X-rays are used. These fundamental differences alter the tumor response and should be incorporated in TRT-specific TCP models. Section 3 describes these differences more in detail, while Section 4 incorporates proliferation and tumor heterogeneity into the TCP model, which are two important radiobiological aspects in both TRT and EBRT.

Next to the clinical relevance of TCP models, their preclinical importance is also highlighted. In the framework of RP development, for example, TCP models can evaluate different combinations of radionuclides and vectors to select the most promising candidates for clinical translation. However, a prerequisite for the correct use of TCP models in preclinical settings is accurate dosimetry. This review discusses the use of dosimetry in preclinical research, with the conclusion that often major assumptions are made or dosimetry is even completely omitted. Nonetheless, Konijnenberg et al. showed the critical dependence of TCP on the dose [2], such that even small variations in absorbed tumor dose can alter the TCP substantially. This means that inaccurate dose estimates can lead to ambiguous results or incorrect dose–response relationships.

## 2. TCP Modelling

Since radiation-induced cell death is a stochastic process, TCP models are essentially statistical models. A commonly used TCP model utilizes the Poisson distribution P(n) to describe the probability for *n* out of $n_{0}$ clonogenic cells to survive a treatment [3]. TCP then corresponds to the chance that none of them survive,

(1) $$\mathrm{TCP}=P\left(n=0\right)=e^{-n_{0}S},$$

where *S* is the chance for a single cell to survive. Next to the Poisson distribution, the Binomial distribution B(n)—which can be regarded as the discrete version of the continuous Poisson distribution—can also be used to describe TCP [4]:

(2) $$\mathrm{TCP}=B\left(n=0\right)={\left(1-S\right)}^{n_{0}}.$$

Both of these models coincide with a large number of clonogenic cells $n_{0}$ and a small cell survival probability *S*, which is the case for typical radiation treatments ($n_{0}\approx 10^{9}$ for 1 cm${}^{3}$ tumor tissue and $S\approx 10^{-1}$ for 2 Gy) [4,5].

**Figure 1:**
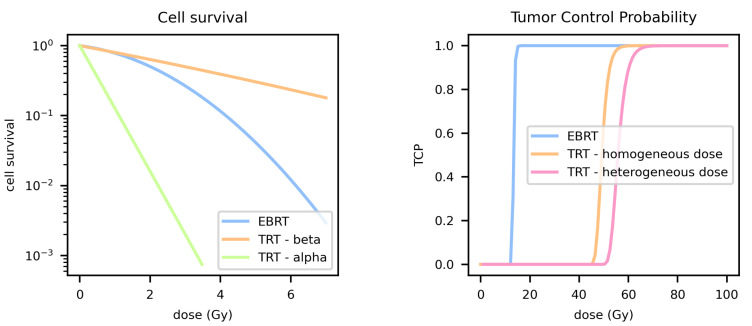
Cell survival and tumor control probability (TCP) curves of external beam radiotherapy (EBRT) vs. targeted radionuclide therapy (TRT), including the comparison between alpha and beta radiation as well as homogeneous vs. heterogeneous dose; while EBRT has a linear-quadratic survival curve, the one of TRT is more linear. Alpha particles are more effective compared to EBRT, while beta particles are less effective. The TCP curves show that the heterogeneous dose distributions decreases the efficacy. Most of the input data are taken from Bernhardt et al., who simulated a metastatic control probability model for prostate cancer [9] ($T_{eff}=51$ h, $T_{r}=1.9$ h, α/β=1.5 Gy, α=0.147 Gy${}^{-1}$ and $N=1.79\times 10^{8}$), combined with the relative biological effectiveness (RBE) values of 1.5 and 14 for ${}^{177}$Lu and ${}^{241}$Am, respectively, for the prostate cancer cell line PC3 [10]. For the dose heterogeneity, a lognormal dose distribution with shape parameter $\sigma_{D}=0.1$ was assumed.

The next step is to find an expression for the probability of a single cell to survive the treatment, i.e., *S* from Equations (1) and (2). A widely accepted cell survival model is the linear quadratic model (LQ) (see Figure 1), for which the strength is to be experimentally confirmed while providing radiobiological insights [6]. The basic LQ model is stated as

(3) $$S=e^{-\alpha D-\beta GD^{2}}.$$

The exponent consists of a linear and quadratic term, with each term representing a distinct type of lethal DNA damage that can induce cell death. As described in detail by Dale et al., lethal DNA damage can either originate from a single ionizing event or from multiple individual sub-lethal ionizing events [7,8]. The latter one requires at least two proximal, independent ionizing interactions to induce lethal cell damage, which results in a quadratic dependence on the dose *D*.

The contribution of the linear part is determined by the radiosensitivity parameter α. It depends on the irradiated tissue characteristics (oxygenation, cell-cycle phase, etc.) as well as on the linear energy transfer (LET) of the radiation. LET is defined as the amount of energy that is deposited per unit of distance, and thus, quantifies the density of the ionisation tracks for a particular type of radiation. High LET radiation is marked by dense ionisation tracks, which will induce more DNA damages. Note that a single radiation event will result in mainly lethal damages, such that high LET radiation will show an increased linear component α. Examples of high LET radiations are alpha particles and Auger electrons. Beta particles and X-rays, on the other hand, are categorized as low LET radiation and are, thus, less damaging.

The quadratic part consists of the β constant together with the Lea-Catcheside or dose-protraction factor *G*. The latter one, *G*, adjusts the quadratic part for the ability of cells to repair sub-lethal damage prior to a second ionization event which is needed to cause lethal damage. This depends on the time scale of the repair mechanisms on the one hand, and the dose rate on the other hand. For an arbitrary dose rate $\dot{D}$ and repair rate μ, the Lea-Catcheside factor is defined as [3]

(4) $$G\left(\dot{D},\mu \right)=\frac{2}{D^{2}}\int_{-\infty }^{+\infty }\dot{D}\left(t\right)dt\int_{-\infty }^{t}\dot{D}\left(t^{\prime }\right)e^{\mu (t^{\prime }-t)}dt^{\prime }.$$

Finally, this factor is multiplied by the constant β, which indicates how sensitive a particular type of tissue is to changes in dose rate or irradiation schemes [7]. However, the ratio α/β is mostly reported, which is high for fast-responding tissues, such as tumor tissue, and low for late-responding tissues.

The radiobiological constants α and β can be accessed by a fitting of Equation (3) to survival curves, which can be provided by in vitro colony survival assays. Herein, the ability of a single cell to grow into a colony is evaluated as the function of the absorbed dose. The resulting parameters depend largely on the dosimetry, which is used to determine the absorbed dose. This is described in detail in Section 5.2. The repair constant μ is required to calculate the Lea-Catcheside factor. This is commonly achieved by looking at the amount of DNA damage over time via γ-H2AX assays, which is complicated for TRT due to protracted DNA damage induction. Forand et al. have shown that after DNA repair, γ-H2AX remains temporarily localized near the initial DNA damage, which should be taken into account [11].

The TCP model can be extended in case of metastatic disease. Herein, multiple tumors of varying size are present and the metastatic control probability (MCP) represents the chance that all *m* metastases are cured [9]:

(5) $$\mathrm{MCP}=\prod_{i=1}^{m}{\mathrm{TCP}}_{i}.$$

## 3. TCP for Targeted Radionuclide Therapy

Radionuclide therapy is characterized by (i) a relatively low dose rate on a protracted timescale that varies over time, (ii) a heterogeneous dose distribution on different levels, and (iii) radiation with various LET, such as high LET alpha particles or Auger electrons and low LET electron radiation, in contrast with EBRT, which uses only low LET X-rays. To what extent these characteristics will affect the TCP depends on the radiation type. Alpha particles and Auger electrons have a very short range compared to beta particles (see Table 1), such that the effect of dose heterogeneity will be much more pronounced. Furthermore, the high LET of the alpha particles and Auger electrons results in mainly lethal DNA damage. Accordingly, the effect of dose rate on the repair of sub-lethal damages will be minimal for high LET radiation, while this is an important factor for low LET beta particles. The following sections will discuss these effects in detail.

### 3.1. Dose Rate

The dose rate within the tumor is relatively low in TRT compared to EBRT. Furthermore, the dose rate changes over time with an initial increase during the uptake phase of the RP, followed by a slow exponential decrease, determined by both the physical decay and the biological half-life of the RP. The low dose rate allows cells to repair the majority of the sub-lethal DNA damage prior to a secondary ionization event. This leads to a reduced dose–response effect. On the other hand, when both tumor and healthy tissues receive a similar dose rate, this may lead to a healthy tissue-sparing effect [8]. As mentioned before, since high LET radiation induces mainly lethal damages, this effect will be most important for low LET radiation, such as beta particles.

Note that the Lea-Catcheside factor describes this phenomenon exactly. Its definition—Equation (4)—requires the dose rate scheme and the repair rate as input. Solanki et al. derived an expression of G for TRT, which includes both the uptake and excretion phase [13]. However, the assumption that the RP is taken up immediately, substantially simplifies the result:

(6) $$G_{TRT}\approx \frac{\lambda_{eff}}{\mu +\lambda_{eff}}.$$

Mu is the repair rate and $\lambda_{eff}$ is the effective decay constant of the RP. When DNA repair is fast compared to the dose rate ($\mu \gg \lambda_{eff}$), then $G_{TRT}\approx 0$, such that the contribution of the quadratic part, depicting lethal damage by superimposed ionization events, will be minimal. The cell survival curve will, therefore, be more linear, as shown in Figure 1. Including the gradual, initial uptake phase of the RP will reduce the Lea-Catcheside factor G even further. Moreover, Solanki et al. found in vitro that a longer uptake phase could even reduce the efficacy during the clearance phase, which might be the result of adaptive tumor responses [13].

Recently, Gholami et al. investigated the effect of dose rate in vitro by comparing both low and high dose rate of ^90^Y TRT with EBRT [14]. They reported a tremendous reduction in the contribution of the quadratic part for reduced dose rates as well as an overall reduced efficacy, as expected. The other way around, Nonnekens et al. were able to block the repair of single cell breaks in the DNA using a PARP inhibitor and observed a significant reduction in cell survival [15]. Unfortunately, no dose was reported, and hence, no α, β and G values could be estimated.

A final remark on dose rate is that at extremely low dose rates, the eradication of clonogenic cells might be in competition with cell proliferation. The critical dose rate at which cell eradication matches cell proliferation, is expressed via

$$R_{crit}=\frac{\ln2}{\alpha T_{p}},$$

where $T_{p}$ is the cell proliferation doubling time and α the cell radiosensitivity [16]. At a certain time, the dose rate during TRT will drop below this critical value, such that tumor cell proliferation will exceed cell death.

### 3.2. Heterogeneous Dose Distribution

**Figure 2:**
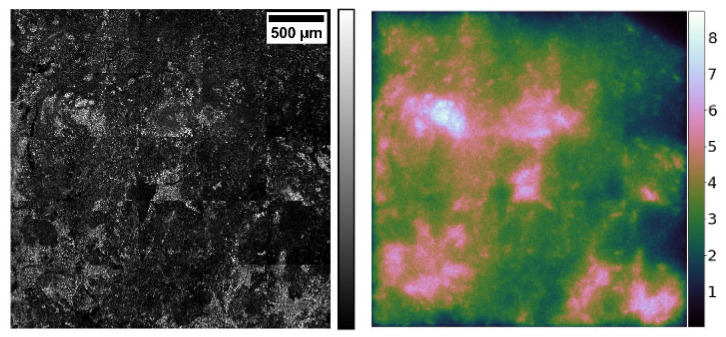
Immunofluorescent staining of receptor expression (somatostatin receptor 2) on a tumor tissue section, showing a heterogeneous receptor expression (**left**), and a corresponding heterogeneous dose distribution in Gray for the beta-emitter ${}^{177}$Lu (**right**). This research was originally published in JNM. [23].

Following administration to the patient, radiopharmaceuticals are distributed throughout the tumor and deliver a dose to the surrounding tumor tissue up to a maximum range, which is specific for each radionuclide. Accordingly, the non-homogeneous distribution of an RP within the tumor can be translated into a non-homogeneous dose distribution (see Figure 2). At the tissue level, this can be observed as an inter-cellular dose heterogeneity. Each cell receives a different dose, and consequently shows a distinct treatment response. At the cellular level, the subcellular pathway of the RP results in an intra-cellular dose heterogeneity as well. Internalization will increase the dose to the cell nucleus for particles with a short range, and since the nucleus is considered as the primary target, this is expected to increase the TCP.

#### 3.2.1. Tumor/Tissue Dose Heterogeneity

On a tissue level, radiopharmaceuticals may be heterogeneously distributed due to a heterogeneous receptor expression and differences in perfusion, where limited perfusion can prevent radiopharmaceuticals from reaching the tumor cells. This results in a heterogeneous dose distribution as well, for which the low and high dose regions can differ by a factor of five, as observed by Timmermand et al. [17]. A heterogeneous dose distribution induces a correlated non-uniform treatment response [18,19]. In order to account for heterogeneity in the TCP model, multiple compartments can be defined, such that the dose $D_{i}$ in each compartment can be considered homogeneous. At a tissue level, such a compartment can be a voxel (i.e., 3D pixel), originating from 3D in vivo imaging techniques or autoradiography images (see Section 5.3), while for in vitro studies, the compartments could refer to single cells. Consequently, Voxel Control Probability ($VCP(D_{i})$) can be assigned to each compartment, representing the probability that this specific compartment will be controlled. The overall TCP is then the probability that all the compartments are controlled:

(7) $$\mathrm{TCP}=\prod_{i}\mathrm{VCP}\left(D_{i}\right).$$

This concept was first used by A.E. Nahum to investigate the effect of a reduced dose at the tumor boundary [20]. More recently, this concept was extended by Uusijärvi et al. [21], who employed hypothetical normal and log-normal activity distributions over the tumor tissue. Both in silico studies found a reduced TCP for a heterogeneous dose distribution compared to an analogue homogeneous distribution, as expected. Repopulation occurs within tumor regions receiving a low dose, which in turn reduces the treatment efficacy. An increased dose barely affects regions with (almost) no radionuclide uptake, hence, it shows saturation in the cell survival curve [22]. In contrast, Tamborino et al. predicted an increased efficacy for a heterogeneous dose distribution based on autoradiography images of in vivo tumor models [23]. They devote this observation to the use of a cell-line with a more homogeneous receptor expression in combination with the beta particle-emitting radionuclide (${}^{177}$Lu), which most likely blurred the effect. This contradiction indicates that more experimental data are required to investigate the effect of a heterogeneous dose distribution on the treatment outcome.

The dose heterogeneity issue could theoretically be overcome by the use of radionuclides with a longer range. This way, regions with low RP uptake could still be irradiated by the so called cross-fire effect of radionuclides further away. However, long-range radiation is less suitable for smaller tumors or metastases since a great fraction of the energy is deposited outside of the tumor [24]. Furthermore, long-range beta electrons are also observed to be less effective compared to short-range Auger electrons and alpha particles due to the difference in LET [25,26]. The metastatic tumor model of Bernhardt et al. shows nicely how each radionuclide has their own optimal tumor volume [9]. Therefore, the optimal radionuclide needs to be selected depending on the tumor size. For metastatic disease, one could utilize a mix of radionuclides to combine the advantages of different types of radiation [27,28].

#### 3.2.2. Subcellular Dose Heterogeneity

Alpha particles and Auger electrons have an extremely short range of 28–100 μm and several nanometers, respectively, [29]. Consequently, their subcellular pathway results in intra-cellular dose heterogeneity. As such, TCP can substantially increase when the absorbed dose to radio-sensitive cell organelles is maximized. The nuclear DNA is considered the primary target since non-repairable DNA damage can result in apoptosis and indeed, many studies have shown a clear nuclear dose–response relationship, as summarized by Bavelaar et al. [30]. RP may be directed towards the nucleus via receptor-medicated internalization, or by the targeting of endosomes, which even increases the efficacy for longer-range radiation, such as ${}^{177}$Lu [31]. Furthermore, differential internalization between the tumor and healthy cells might, therefore, increase the therapeutic index.

The previous paragraph applies for alpha particles and beta particles with a short range. However, the range of Auger electrons is too small to target the nuclear DNA from inside the cytoplasm. Nonetheless, Auger-emitting particles have shown to be effective from inside the cytoplasm. A study by Feudenberg et al. clearly suggested the existence of extra-nuclear targets for Auger electrons [32], such as the mitochondria and cell membrane. Damage to these targets may induce cell death, though with less sensitivity [30]. Recent findings even suggest that Auger emitters coupled to cell membrane-targeting antagonists might be more potent than internalizing or nuclear targeting agonists [33]. However, these findings could also be the result of the overall higher tumor uptake of the antagonists. Therefore, absorbed doses to the main cell compartments and/or organelles (nucleus, cytoplasm, cell membrane) need to be estimated accurately before any conclusion can be drawn from these results.

### 3.3. Linear Energy Transfer

The third aspect for modeling response in TRT is the radiation quality. In EBRT, the dose is delivered via X-ray photons, while TRT exploits the short-range alpha and beta radiation as well as Auger and Conversion electrons. Besides the specific range of each radiation type, they also distinct themselves by the means of their LET. As explained in Section 2, the more effective DNA damage induced by higher LET radiation results in an increase in the contribution of the linear α component. This can be observed by the more steep and straight survival curve for higher LET radiation [3].

The relative biological effectiveness (RBE) quantifies this effect by evaluating the dose from the investigated high LET radiation *D* that is required to obtain the same biological endpoint (e.g., cell survival) as a dose $D_{ref}$ from a reference low LET radiation:

(8) $$\mathrm{RBE}=\frac{D_{ref}}{D}.$$

However, this is a variable quantity as it depends on the selected reference dose, the biological endpoint, and on the α/β ratio of the tissue [34]. Claesson et al. showed that the RBE is also affected by the cell-cycle phase and reported an RBE variation between 1.8 and 8.6 [35]. In order to exclude the dependence on the reference dose, Dale and Jones introduced the intrinsic RBE${}_{m}$, which is defined as the RBE at a zero dose, such that it reflects the ratio of the initial slopes of the survival curves, and thus, the ratio of the α-components of the LQ model [34]. This concept can be used to include the radiation quality in the TCP model via

(9) $$\alpha =\alpha_{ref}\cdot {\mathrm{RBE}}_{m},$$

where $\alpha_{ref}$ is the radiosensitivity for the reference radiation, which only depends on the tissue.

It should be noted that experimentally evaluating the RBE is not an obvious task. The cell survival curves for each radiation type, as well as the reference radiation, requires highly accurate dosimetry in order to extract the α and β values. In Section 5.2, 28 reported survival curves in the function of dose for TRT are evaluated. For those who compared the response of TRT to EBRT, the RBE${}_{m}$ was calculated via Equation (9) and reported in Table 2. Unfortunately, large variations in RBE were observed, resulting in substantial standard deviations, and thus, meaningless results. Therefore, these values need to be interpreted with care.

## 4. Extended Modelling

The previous section focused on the specific characteristics of radionuclide therapy, such as dose rate, dose heterogeneity, and LET. However, radiobiological characteristics that are relevant to radiation therapy in general, can be implemented into a TCP model as well. Three important aspects are tumor proliferation or repopulation, tumor heterogeneity, and bystander effects.

### 4.1. Repopulation

During the treatment, the irradiated tumor will ideally start to shrink because of the extended cell death. As a reaction to this shrinkage, an increase in the proliferation rate has been observed, i.e., the repopulation of a tumor [3]. At the time this repopulation is initiated, treatment is generally still ongoing. Consequently, a higher dose is required to kill the additional cancerous cells.

Repopulation can be incorporated into the Poisson TCP model by adding an exponential growth term to the cell survival equation

(10) $$\begin{array}{ccc} S=e^{-\alpha D\;-\;\beta GD^{2}}e^{\ln2\frac{\left(T\;-\;T_{k}\right)}{T_{p}}} & \; & T>T_{k} \end{array}$$

(11) $$\begin{array}{ccc} \;S=e^{-\alpha D\;-\;\beta GD^{2}} & \; & T\leq T_{k}. \end{array}$$

$T_{p}$ is the proliferation doubling time, *T* is the overall treatment time, and $T_{k}$ is the off-set time for repopulation to initiate [4]. A drawback of the Poisson TCP model is the lack of time-dependency. For this reason, Zaider and Minerbo introduced an analytical expression for the TCP model with explicit time-dependency [42]:

(12) $$\mathrm{TCP}\left(t\right)={\left[1-\frac{S\left(t\right)e^{(b\;-\;d)t}}{1+bS\left(t\right)e^{(b\;-\;d)t}\int_{0}^{t}\frac{dt^{\prime }}{S\left(t^{\prime }\right)e^{\left(b\;-\;d\right)t^{\prime }}}}\right]}^{n_{0}}.$$

This formula is derived from a differential equation that expresses the change in the number of clonogenic cells as the function of natural formation *b* and death *d* rates, combined with the radiation-induced cell killing rate. The parameter *S* refers to cell survival and *n* represents the initial number of clonogenic cells. A time-dependent cell formation rate should be introduced to include an off-set for repopulation. Note that this model will result in a higher TCP compared to the time-independent expression proposed in Equation (10), and in case b=d=0, this expression will be reduced to the binomial TCP model (Equation (2)).

### 4.2. Heterogeneous Dose Response

A heterogeneous dose response should not be confused with dose heterogeneity, although both have an impact on TCP. In addition to dose heterogeneity, each tumor cell can also respond differently to the absorbed dose for various reasons. In regions with lower vascularisation, cells are more hypoxic, and since oxygen can act as a radiosensitiser, those cells may be more radio-resistant. Alpha particles and Auger electrons, however, are shown to be less affected by hypoxia [43]. The radiosensitivity also varies throughout the cell cycle due to the availability of repair mechanisms, cell cycle checkpoints, mitotic catastrophe, etc. [6]. Dawson and Hillen extended the time-dependent Zaider and Minerbo TCP model to include this cell-cycle dependency [44]. Herein, the clonogenic cells were divided into a quiescent (G0) and active (G1, S, G2, M) phase, with cell-cycle phase-specific radiosensitivity. Cells can transfer from one phase to another over time, and more compartments can be included if required.

Next to heterogeneous radiosensitivity, tumor cell density is variable as well. A higher cell density requires a higher dose to account for additional clonogenic cells. However, heterogeneous cell density has less impact on the TCP compared to heterogeneities in radiosensitivity [45].

It is important to consider tumor heterogeneity in a TCP model since a relapse can occur from a niche of radio-resistant cells or from the more dense tumoral subregions [1,42]. One way to do this is via voxel control probabilities, similar to Equation (7) for the dose heterogeneity. The global TCP is given by

(13) $$\mathrm{TCP}=\prod_{i}\mathrm{VCP}\left(\alpha_{i},\rho_{i},D_{i}\right).$$

In the case of heterogeneous cell density, this model shows that the maximal TCP is achieved with a homogeneous VCP distribution [45]. However, this is not the case for variations in radiosensitivity. In practice, the individual voxel radiosensitivies $\alpha_{i}$ and densities $\rho_{i}$ can be obtained via various imaging methods [1]. Although, a simplified model is often required because of the limitations of imaging. To simplify the model, one can split the tumor in oxic and hypoxic regions to account for the differences in radiosensitivity between an oxic and hypoxic tumor environment. Another possibility is to introduce a probability distribution for radiosensitivity. Modeling cell survival according to a Gaussian distribution centered around an average α value with a variance of $\sigma_{\alpha }^{2}$ results in [46]

(14) $$S=e^{-\alpha D-\left(\beta \;-\;\frac{1}{2}\sigma_{\alpha }^{2}\right)D^{2}}.$$

Note that the additional positive term $\frac{1}{2}\sigma_{\alpha }^{2}D^{2}$ increases cell survival, and thus, decreases TCP as a consequence of the more radio-resistant cells which have a larger impact on therapy efficacy compared to the more radio-sensitive cells (see Figure 1).

Ideally, this heterogeneous dose response should be correlated with the dose heterogeneity discussed in Section 3.2.1 to make sure that intratumoral regions with a high absorbed dose overlap with the more radio-resistant tumoral subregions. Unfortunately, those are generally inversely correlated because of perfusion [47]. Regions with poor perfusion are more hypoxic, and thus, more radio-resistant, as explained above, while at the same time, those regions will have a lower radiopharmaceutical uptake, and thus, receive a lower absorbed dose.

### 4.3. Bystander Effect

The bystander effect refers to the phenomenon where non-irradiated cells show a dose–response relation due to cell-signaling from distantly irradiated cells, which is a well-recognized effect in EBRT. Due to the cross-fire effect, which is inherent to TRT and induces similar effects in distant cells, studying the bystander effect for TRT is more complicated [48]. Nonetheless, bystander effects could be of large importance in TRT since it can induce effects in low dose regions, and thereby, reduce issues of dose-heterogeneity [22]. To our knowledge, TCP models that incorporate the bystander effect have not been established so far.

## 5. Preclinical Dosimetry

While the administered activity is a more practical quantity to evaluate the treatment outcome, a more relevant parameter for evaluating radiobiological effects is the absorbed dose, amongst other important parameters such as dose rate, LET, heterogeneity, and radiobiological parameters. Estimating the absorbed dose from an administered activity is unfortunately not straightforward and accurate data should be gathered regarding the RP uptake and distribution, and cell/tissue geometries, combined with dose-deposition simulations. Due to its complexity, dosimetry is often omitted in preclinical research, or major assumptions are made, which results in substantial uncertainties.

These uncertainties might have a major impact on the interpretation of the observed effects, given that the TRT outcome depends on the absorbed dose. Konijnenberg et al. have shown that a commonly observed dose uncertainty of around 10% can result in differences in treatment outcome, ranging from a limited response (TCP = 1%) to almost complete remission (TCP = 98%) [2]. This points out the necessity for accurate preclinical dosimetry to correctly determine dose-effect relationships and to allow an objective and proper comparison of multiple treatments, both in vitro and in vivo.

### 5.1. MIRD Scheme

The Medical Internal Radiation Dose (MIRD) Committee proposed the following scheme for calculating the absorbed dose *D* in a target region $r_{T}$ from activity located in multiple source regions $r_{S}$ [49]:

(15) $$D\left(r_{T},T_{D}\right)=\sum_{r_{S}}\tilde{A}\left(r_{S},T_{D}\right)S\left(r_{T}\leftarrow r_{S}\right).$$

The definition of these regions is specific for each situation. For in vivo studies, these regions refer to whole organs or small voxels, while in the case of in vitro models, they refer to single cells or cell organelles. For each source region, the time-integrated activity $\tilde{A}$ is multiplied by the S-value. The former one represents the total number of radioactive decays within that source region $r_{S}$ over the treatment time $T_{D}$, i.e., the activity integrated over time. The latter one is defined as the mean dose absorbed by the target region $r_{T}$ per radioactive decay in the source region $r_{S}$. This factor depends on the specific geometry of the source and target region as well as on the radiation characteristics of the radionuclide. How this general scheme is applied in specific in vitro and in vivo situations is described in the subsequent sections.

### 5.2. In Vitro Dosimetry

**Figure 3:**
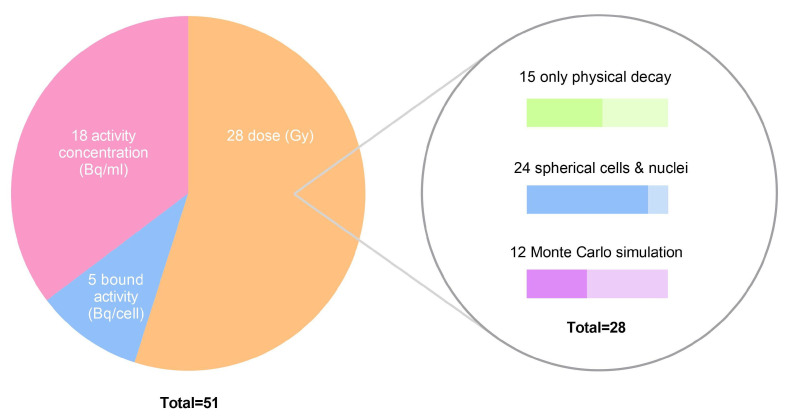
An overview of the evaluated dosimetry for cell survival curves. Fifty-one articles reporting a survival curve for TRT were evaluated. The survival curves were reported as a function of administered activity, bound activity or absorbed dose. The applied dosimetry often goes along with approximations such as neglecting biological decay, assuming spherical cells and nuclei, or applying simplified dose-deposition simulations instead of detailed Monte Carlo simulations.

In vitro studies are generally performed in the first stages of RP development and are the preferred method for determining radiobiological parameters and investigating biological mechanisms. However, the experimental conditions can vary substantially, which—in an ideal world—would require for the dosimetry to be repeated for each assay. An example of a standard radiobiological assay is the clonogenic survival assay. This assay generates the tumor cell survival curve, which provides important information on radiobiological parameters, including α and β, when presented as the function of absorbed dose. A PubMed search resulted in 51 papers reporting a survival curve for radionuclide therapy. A schematic overview is given in Figure 3. Eighteen of these studies (35%) did not perform dosimetry at all, and reported survival in the function of administered activity [15,33,50,51,52,53,54,55,56,57,58,59,60,61,62,63,64,65]. However, Freudenberg et al. demonstrated how this can lead to a misinterpretation of the results [66]. Plotting the cell survival in the function of administered activity suggests a difference in effectiveness between various treatments, while a similar effectiveness was observed after applying microdosimetry.

Five studies (10%) reported cell survival in the function of the bound activity per cell [67,68,69,70] or against the time-integrated activity $\tilde{A}$ [71]. Taking this approach avoids issues regarding cell saturation, making $\tilde{A}$ a more relevant reference compared to the administered activity. However, to obtain the actual absorbed dose, one needs to still apply the appropriate S-values. Approximately half of the studies, or 28 (55%) to be more specific, did perform dosimetry and reported cell survival as a function of the average absorbed dose by the tumor cells, or more often, as a function of the absorbed dose by the cell nucleus. However, the accuracy of the applied dosimetry methods is debatable, as often strong assumptions are made on the cell geometry (e.g., spherical cell geometry), RP distribution (e.g., uniform), RP clearance (e.g., no biological clearance) etc.

#### 5.2.1. In Vitro Source Regions

The standard source regions in in vitro assays are the medium (=non-specific dose), the neighboring cells (=cross dose), and the cell organelles in the target cell (=self dose). Typically, the cell organelles to be considered are the cell membrane, the cytoplasm and the nucleus. Although activity uptake in the cell nucleus is limited for most vectors [19,32], Falzone et al. have shown that even little uptake in the nucleus can induce a substantial increase in TCP, especially for particles with a short range, such as Auger electrons [72]. The available methods and techniques to access the subcellular activity distribution in vitro were recently reviewed by Costa et al. [73]. The most common method by far is fractionation, in which the different physiochemical properties of the cell organelles of interest are exploited to obtain separated fractions, which subsequently can be measured with a gamma counter. Although fractionation is a fast, relatively easy and low-cost method, it provides only a population-averaged distribution [73]. In all 27 studies using dosimetry methods in this review, the activity is assumed to be uniformly distributed within the cells. Nonetheless, both flow-cytometry results and autoradiogarphy images showed that the RP uptake follows a log-normal instead of uniform distribution [74,75]. Rajon et al. showed with a computer model how such a log-normal distribution reduces the efficacy of a treatment [76].

#### 5.2.2. Time-Integrated Activity $\tilde{A}$

In order to access the time-integrated activity $\tilde{A}$, and thus, the time-activity curve $A(r_{S},t)$, the activity in each source region needs to be quantified at well-selected time-points. Sufficient time-points are required in order to fit the initial uptake and subsequent clearance phase accurately. Guirriero et al. investigated the effect of using different sets of time-points and fitting models [77]. They highlighted the importance of including late time-points (>2 $\times \;T_{1/2,eff}$) and pointed out the inaccuracy of using mono-exponential clearance models. Additionally, Sefl et al. showed that neglecting the initial uptake phase will lead to a substantial overestimation of the absorbed dose, and thus, an underestimation of cell survival [78]. Nonetheless, 15 out of the 27 studies reporting survival vs. dose (52%) evaluated the RP uptake at only a single time-point and neglected biological decay, which can result in an overestimation of the absorbed dose [10,14,35,37,38,39,41,66,79,80,81,82,83,84,85].

#### 5.2.3. S-Value

S-values should be determined for each cell-type, radionuclide, and experimental set-up. Goddu et al. tabulated S-values for self-dose for various combinations, which were used in 30% of the studies performing dosimetry, whether or not extended by medium-dose simulations [86]. Later on, a Java-applet MIRDcell was developed to calculate both self and cross dose S-values. Both Goddu and MIRDcell use a spherical geometry for the cells, combined with energy deposition based on the continuously slowing down approximation [87]. The latter one is a semi-analytical method that neglects the finite range of secondary electrons and does not take into account straggling effects [88]. At the sub-micron scale (i.e., subcellular level), it was shown that these approximations become inadequate, resulting in S-values which can differ by a factor of two compared to S-values obtained with Monte Carlo simulations, where a collision-by-collision approach is considered [89]. These simulations require appropriate Monte Carlo particle transportation codes such as Geant4, MCNP, PENELOPE or GATE. Twelve authors (43%) did use such a code to compute the detailed dose deposition [14,19,31,32,36,37,40,66,79,80,90,91].

Next to the computational methods that have been used, the geometry of the cell has also a large influence on the S-value. Twenty-four authors (86%) assumed concentric spherical cells and nuclei, despite the fact that cells are adherent during a colony survival assay, and thus, far from spherical. It has been demonstrated that a non-spherical cell [31] or an eccentric nucleus [92] may impact the absorbed dose significantly for short-range radiation. However, realistic cell geometry models are rather complex to implement. A first improvement on the standard concentric geometry was the usage of ellipsoids or oblate spheroids as an approximation for the cells and their nuclei, which was done by Steffen, Yard and Ruigrock [81,91,93]. An even more realistic approximation was made by Tamborino et al, who used the average S-value of nine detailed polygonal mesh cell models based on confocal microscopy images [31]. However, the S-values for those nine models, representing cells within a fixed cell line, showed a large variation. Finally, a completely different approach was taken by Palmer et al., who constructed distance probability density functions (PDF) based on cell culture images [94]. For each PDF bin, a dose-point kernel needs to be applied only once, which substantially reduces the computational time.

For the cross dose, the distribution of cells within a culture plate is of more importance than the geometry of the cells. Generally, it is assumed that the cells are uniformly distributed, although some cell types tend to grow in clusters. Marcatili et al. developed a dosimetry framework for colony survival assays, including random cluster formation [95].

### 5.3. In Vivo Dosimetry

In vivo studies with appropriate animal models allow us to investigate a tumor microenvironment, which is much more representative for the pathology in patients compared to in vitro assays. Organ-level activity distributions can be determined by either ex vivo biodistribution studies or in vivo nuclear molecular imaging techniques.

For the ex vivo approach, animals are sacrificed, organs of interest are collected and subsequently weighted, while organ activities are measured with a gamma counter. This procedure provides accurate activity concentrations for each organ. However, to determine the time-integrated organ and tumor activities, multiple animals need to be sacrificed at different time-points to generate population-averaged time-activity curves for the tumor and organs of interest. Since a single time-activity curve is based on the data of different animals, variability between animals induced by a different disease state or different tumor growth rate can have a significant impact on the final extracted time-activity curve, and thus, on the dosimetry estimates which should, therefore, be interpreted with care. Moreover, ex vivo biodistribution studies do not provide any information on the intra-organ or intra-tumoral activity distribution, such that the effect of heterogeneous activity distributions cannot be taken into account.

On the other hand, autoradiography images are able to provide more detailed information about dose-distribution heterogeneities. Herein, the alpha or beta particle emissions from 2D cryosections are imaged with a high resolution and provide quantitative activity distributions with a resolution up to 15 µm [96]. The trade-off for these high resolution images is the missing 3D information, which limits the dosimetry. It is often assumed that the adjacent sections have identical activity distributions [17,97]. The recovered 3D activity map can then be convoluted with a dose-point kernel (DPK) or a direct Monte Carlo simulation can be performed in order to obtain the dose distribution. As an alternative to these autoradiographic images, immunofluorescent staining of the receptor also provides information on the activity distribution and has been used for dose calculations as well [23]. Although this provides valuable information for the receptor heterogeneity, this does not take into account the heterogeneity of the RP distributions, for example, due to poor perfusion.

Meanwhile, in vivo molecular imaging techniques allow a non-invasive, quantitative assessment of the biodistribution of radiopharmaceuticals, such that animals can be imaged multiple times to generate individual time-activity curves. As such, dosimetry estimates will be much more consisten, while therapy response can be monitored by a longitudinal assessment of the tumor growth within the same animal. The most frequently used molecular imaging technique for dosimetry is Single Photon Emission Tomography (SPECT). This technique is also frequently used for dosimetry in clinical TRT, and was adapted for the smaller dimensions of mice and/or rats to provide adequate imaging performance for preclinical research. Consequently, in vivo preclinical SPECT imaging for dosimetry is also much more similar to the imaging approach used in clinics, and therefore, much more translatable. More specifically, preclincal, or microSPECT (µSPECT), is based on the detection of co-emitted gamma photons by the radionuclides and converts these detected gammas into a quantitative 3D activity distribution via iterative reconstruction algorithms [98]. An additional Computerized Tomography (CT) scan provides anatomical information and allows to perform non-uniform attenuation and scatter correction, which improves the quantitative properties of the SPECT image. The CT scan is also beneficial for identifying the volumes of interest (VOI) in which the total activity concentration is determined. In addition, voxel activities reveal the heterogeneities on the sub-organ level. These are directly available from the quantitative µSPECT/CT images, although the heterogeneity should be differentiated from the noise of the images itself [99]. The voxel activities are limited by the resolution of SPECT images as well, which range from 5–25 mm for clinical SPECT [98]. However, the dimensions of small animals require an even better resolution. This is achieved by the use of multi-pinhole collimators, resulting in a resolution of 1.5 mm [100]. Those voxel activities should be converted to a voxel dose distribution. No tabulated voxel S-values are present for animal models, as is the case for clinical applications [101]. An alternative is the integration of DPK over the voxels with a CPU-intensive conversion of spherical to Cartesian coordinates [102]. This still does not take into account the heterogeneities in tissue density, thus, requiring Monte Carlo simulations. Unfortunately, these require large computation times, which should be considered given the specific inaccuracy of DPK’s.

For both in vivo and ex vivo methods, the conversion of the organ activities into absorbed doses requires organ S-values. These are available from software such as MIRDcalc, OLINDA and IDAC-dose, based on reference phantoms, or can be obtained via Monte Carlo simulations in combination with available digital animal models such as MOBY and ROBY [103].

To conclude, all these techniques are very complementary. Although the activities determined with µSPECT are less accurate compared to ex vivo gamma counter measurements, µSPECT allows to perform longitudinal studies within individual animals and enables a translation to clinical workflow. On the other hand, a single SPECT voxel has dimensions in the order of several mm${}^{3}$, such that activity can still be non-homogeneously distributed within one voxel. Meanwhile, ex vivo autoradiography images and in vitro dosimetry methods are able to provide valuable information regarding dose heterogeneity on a subvoxel and subcellular level, respectively. However, in in vivo studies, a homogeneous dose within each voxel needs to be assumed, since dose distributions at a voxel level are the highest achievable detail. On the other hand, subvoxel heterogeneities can clearly affect the treatment response, which needs to be somehow incorporated in in vivo TCP models. A challenging task is, thus, to translate the effects of a subcellular and subvoxel dose heterogeneity up to the in vivo level.

## 6. Conclusions

Although the extension of TCP models towards TRT has been established quite well from a theoretical viewpoint, sufficient confirmatory experimental data are still lacking. The low and protracted dose rate is understood to decrease the TCP, which can be quantified by the Lea-Catcheside factor. However, to our knowledge, the study by Tamborino et al. is the only study so far to actually include this factor in in vivo TCP models [23]. In addition, the heterogeneous dose distribution, which is inherent to TRT, is expected to decrease the TCP, although prior research data are inconclusive due to contradictory results. The expected lower TCP, due to dose heterogeneity, could be partially compensated by the bystander effect, which, however, is not yet fully understood for TRT and is actually quite hard to include in a TCP model since such a model has not been described so far. Moreover, the effect of the radiation quality, which should also be part of a TCP model, is quantified by RBE, which is a challenging quantity to be determine experimentally, and therefore, not currently accurate enough to actually be used in TCP models. In conclusion, additional comprehensive studies at the sub-cellular, cellular, and organ level are essential to experimentally confirm the theoretical basis of TCP models for TRT and to gain further insight into TRT-specific quantities of these models.

## Figures and Tables

**Table 1:** Overview of the radiation types used in targeted radionuclide therapy. For each radiation type, a specific example is given together with the energy, the range, and the linear energy transfer (LET) of the emitted particles [12].

Radionuclide	Radiation Type	Energy	Range	LET
^90^Y	beta	50–2300 keV	0.05–12 mm	0.2 keV/mm
^111^In	Auger	eV–keV	2–500 nm	4–6 keV/mm
^225^Ac	alpha	5–9 MeV	40–100 μm	80 keV/mm

**Table 2:** Overview of the RBE${}_{m}$ obtained via Equation (9) for the 28 survival curves in the function of absorbed dose discussed in Section 5.2. For each RBE${}_{m}$, details are given about the radionuclide, the cell type, and the applied dosimetry (physical or effective decay for the Time Activity Constant (TAC), the cell geometry, and the target region). The results are averaged for beta, Auger, and alpha radiation, and the standard deviation is reported.

Radionuclide	Cell Line	TAC	Cell Geometry	Target	RBE_*m*_	Ref.
${}^{131}$I	CA20948	$T_{eff}$	sphere	entire cell	1.2	[36]
${}^{177}$Lu	U2OS + SSTR2	$T_{eff}$	realistic	nucleus	1.7	[31]
${}^{177}$Lu	U2OS + SSTR2	$T_{eff}$	sphere	nucleus	0.5	[19]
${}^{177}$Lu	CA20948	$T_{eff}$	sphere	nucleus	3.5	[19]
${}^{177}$Lu	CA20948	$T_{ph}$	sphere	entire cell	0.4	[37]
${}^{177}$Lu	Capan-2	$T_{ph}$	sphere	nucleus	1	[38]
${}^{177}$Lu	LNCaP	$T_{ph}$	sphere	entire cell	0.4	[10]
${}^{177}$Lu	DU145	$T_{ph}$	sphere	entire cell	1.5	[10]
${}^{177}$Lu	PC3	$T_{ph}$	sphere	entire cell	1.5	[10]
${}^{32}$P	SCL-II	$T_{ph}$	sphere	nucleus	0.5	[39]
${}^{90}$Y	HCT116	$T_{ph}$	sphere	nucleus	0.2	[14]
${}^{90}$Y	SW48	$T_{ph}$	sphere	nucleus	0.2	[14]
${}^{90}$Y	HT29	$T_{ph}$	sphere	nucleus	0.2	[14]
Average RBE${}_{beta}$					1.0 ± 0.9	
${}^{123}$I	PC Cl3	$T_{eff}$	sphere	nucleus	3.4	[32]
${}^{123}$I	PC Cl3	$T_{eff}$	sphere	cytoplasm	1.7	[32]
${}^{123}$I	PC Cl3	$T_{eff}$	sphere	entire cell	1.9	[32]
${}^{99m}$Tc	PC Cl3	$T_{eff}$	sphere	nucleus	2.2	[32]
${}^{99m}$Tc	PC Cl3	$T_{eff}$	sphere	cytoplasm	0.7	[32]
${}^{99m}$Tc	PC Cl3	$T_{eff}$	sphere	entire cell	0.8	[32]
${}^{125}$I	SCL-II	$T_{ph}$	sphere	nucleus	4.5	[39]
${}^{64}$Cu	MCF7/HER2-18	$T_{eff}$	sphere	nucleus	0.6	[40]
Average RBE${}_{Auger}$					2.0 ± 1.3	
${}^{213}$Bi	CA20948	$T_{ph}$	sphere	entire cell	2.7	[37]
${}^{213}$Bi	CA20948	$T_{ph}$	sphere	entire cell	3.5	[37]
${}^{213}$Bi	Capan-2	$T_{ph}$	sphere	nucleus	3.4	[38]
${}^{241}$Am	LNCaP	$T_{ph}$	sphere	entire cell	8.1	[10]
${}^{241}$Am	DU145	$T_{ph}$	sphere	entire cell	15.2	[10]
${}^{241}$Am	PC3	$T_{ph}$	sphere	entire cell	14.0	[10]
${}^{241}$Am	SW-1573	$T_{ph}$	sphere	nucleus	14.7	[41]
Average RBE${}_{alpha}$					8.8 ± 5.3	

## Data Availability

Not applicable.
